# Paradigm shift in the diagnosis of peste des petits ruminants: scoping review

**DOI:** 10.1186/s13028-020-0505-x

**Published:** 2020-01-29

**Authors:** Edson Kinimi, Steven Odongo, Serge Muyldermans, Richard Kock, Gerald Misinzo

**Affiliations:** 1 SACIDS Africa Centre of Excellence for Infectious Diseases of Humans and Animals in East and Southern Africa (SACIDS-ACE), SACIDS Foundation for One Health, Sokoine University of Agriculture, P.O. Box 3297, Morogoro, Tanzania; 2 Department of Biotechnical and Diagnostic Sciences, College of Veterinary Medicine, Animal Resources and Biosecurity (COVAB), Makerere University, P.O. Box 7962, Kampala, Uganda; 3 Department of Cellular and Molecular Immunology, Vrije Universiteit Brussel, Pleinlaan 2, 1050 Brussels, Belgium; 4 The Royal Veterinary College, University of London, Hawkshead Lane, North Mymms, Hatfield, Hertfordshire AL9 7TA UK

**Keywords:** Diagnostics, Nanobodies, Nanopore, *Peste des petits ruminants*

## Abstract

*Peste des petits ruminants virus* causes a highly contagious disease, which poses enormous economic losses in domestic animals and threatens the conservation of wild herbivores. Diagnosis remains a cornerstone to the Peste des petits ruminants Global Control and Eradication Strategy, an initiative of the World Organisation for Animal Health and the Food and Agriculture Organisation. The present review presents the peste des petits ruminants diagnostic landscape, including the practicality of commercially available diagnostic tools, prototype tests and opportunities for new technologies. The most common peste des petits ruminants diagnostic tools include; agar gel immunodiffusion, counter-immunoelectrophoresis, enzyme-linked immunosorbent assays, reverse transcription polymerase chain reaction either gel-based or real-time, reverse transcription loop-mediated isothermal amplification, reverse transcription recombinase polymerase amplification assays, immunochromatographic lateral flow devices, luciferase immunoprecipitation system and pseudotype-based assays. These tests vary in their technical demands, but all require a laboratory with exception of immunochromatographic lateral flow and possibly reverse transcription loop-mediated isothermal amplification and reverse transcription recombinase polymerase amplification assays. Thus, we are proposing an efficient integration of diagnostic tests for rapid and correct identification of peste des petits ruminants in endemic zones and to rapidly confirm outbreaks. Deployment of pen-side tests will improve diagnostic capacity in extremely remote settings and susceptible wildlife ecosystems, where transportation of clinical samples in the optimum cold chain is unreliable.

## Background

*Peste des petits ruminants virus* (PPRV) causes an acute and highly contagious infection, which can cause significant socio-economic losses in domestic animals and threatens the conservation of wild herbivores. The PPRV belongs to the genus Morbillivirus of the family *Paramyxoviridae* [1, 2], which includes eradicated *Rinderpest virus, Measles virus*, *Canine distemper virus*, *Phocine distemper virus*, Cetacean and Feline morbilliviruses [3]. Rapid field diagnostics against rinderpest became available in final phases of the eradication process, but were never really tested within livestock whilst proving valuable in wildlife environment [4–6]. The rinderpest eradication in 2011 provided the pathway and the possibility of peste des petits ruminants (PPR) eradication given the close phylogeny of these viruses, availability of a reliable and effective vaccine against PPR and sensitive and specific diagnostic tests [7]. PPR most likely emerged in the early part of the twentieth century whilst its presence was masked by ongoing rinderpest epidemics, which also affected small stocks and where immunity was cross protective [8]. Based on phylo-geographical analysis, geographic origins of the most recent common ancestor of PPRV lineages I, II, and III were proposed to originate from Africa whilst lineage IV might have originated from India [9]. PPRV has continued to expand its geographic boundaries, reaching regions previously not infected and putting hundreds of millions of both domestic small ruminants and wildlife at risk of infection. However, the occurrence of PPRV in previously uninfected regions, together with the mixing of lineages in endemically infected countries, highlights the dynamic and transboundary nature of this disease [10–12]. The expansion of PPR range has been known for many decades but it has taken a considerable time to raise international interest and to bring PPR to the status of a priority disease for livelihood and food security. PPR was finally included in the Global Framework for the Progressive Control of Transboundary Animal Diseases (GF-TADs), an initiative of the World Organisation for Animal Health (OIE) and the Food and Agriculture Organisation (FAO) of the United Nations [13, 14]. The annual global impacts of PPR was in 2017 estimated at between US$1.4 and $2.1 billion [13]. However, it is estimated that an investment of US$ 7.1 billion on global PPR eradication could be recovered within 5 years of successful eradication [13]. Some academics believe that the actual cost of eradication could be much lower than this [15], but unfortunately the tardiness of the response to its expansion, increases the likely cost by the day.

Reported PPR outbreaks and infection studies in captive and wild ruminants have extended the known spectrum of potentially infected species to include most *bovidae* and *suidae*. In 2014, there were warnings of the risk of PPR infection of the saiga antelope (*Saiga tatarica mongolica*), which is a critically endangered species in central Asia [16], and since then there has been no proper actions to protect susceptible wild ruminants population from PPR outbreaks. This was followed by PPR epidemic in the small surviving population of a sub-species of saiga with extinction of more than half of the population [17]. Consequently, the saiga catastrophes emphasized the failure of PPR eradication strategies in considering wildlife and possible virus spill over from livestock. From 2014 to 2016, more than 1000 wild goats (*Capra aegagrus*) and sheep (*Ovis orientalis*) in the northern and central provinces of Iran died from PPRV infection [18]. Transmission of PPRV from infected goats to cattle has also been reported [19], and PPRV antigen has been detected in camels [20] and even companion animals, in particular dogs [21]. Goats and sheep are the maintenance hosts and the other hosts are apparently considered as spill over without any other reservoir populations confirmed.

Sheep and goats are vital for more than 330 million poor subsistence and marginal farmers in Africa and Asia a home to more than 1.7 billion sheep and goats, where over 80% of the world’s small ruminants occur and here PPR causes food insecurity and contributes to poverty [13, 22]. The clinical signs elicited by PPRV may vary depending on the breed of the affected animal species and/or the strain of virus [23, 24]. Besides, other factors such as the resilience of the population, nutrition, co-infection and other stressors contribute to the pathogenesis of PPRV infection. The severity of disease depends on the immune status of the animal; for example, newborn animals become susceptible to PPRV infection at three to 4 months of age following natural decline in colostral antibodies. Early and accurate diagnosis of PPRV infection is important for prompt control and this can be facilitated by pen-side diagnostics (Table 1). The availability of simple cost-effective pen-side diagnostics and laboratory based tests would aid in the prevention and control of PPR in low-income countries. However, these tests should be performed in reference with OIE prescribed tests for confirmation of clinical cases using immunocapture enzyme-linked immunosorbent assay (IC-ELISA) and reverse transcription polymerase chain reaction (RT-PCR) and for certification of population freedom from infection by competitive enzyme-linked immunosorbent assay (C-ELISA) and virus neutralization test (VNT) [25] (Table 2). Thus, the use of automated assays that do not require supplementary multiple reagents and lateral flow diagnostic strips technologies based on low cost immunoreagents such as nanobodies may accelerate the development of powerful diagnostic assays. In addition, lack of validated tests amongst wildlife species creates uncertainties in the interpretation of surveillance data.

**Table 1:** Diagnostic value of commercially available field-deployable diagnostic tools and pen-side prototype tests for PPR diagnosis

Diagnostic tests	Target (s)	Merits	Limitations	Detection limit	References
Immunochromatography lateral flow test	H and N proteins	Very rapid and pen-side test	Less sensitive than PCR	10^3^ to 10^4^ TCID_50_	[26]
Quantum dots lateral flow	PPRV IgG antibodies	Ultrasensitive and field test	Cannot detect active case	Specificity 99.47%, sensitivity 97.67%	[27]
One-step RT-LAMP	M gene	Rapid and easy to perform	Not a field-level diagnostic	1.41 × 10^−4^ ng total RNA per assay	[28]
Two-step RT-LAMP	N gene	Rapid and pen-side test	Require six primers	100% specificity and sensitivity	[28–30]
Recombinase polymerase amplification assay	N gene	Rapid compared to RT-LAMP	Less sensitive compared to RT-PCR	Sensitivity 90% and specificity100%	[31, 32]
Oxford nanopore MinION sequencers	Viral genome	Rapid	Prone to high host nucleic acids		[33, 34]

**Table 2:** OIE diagnostic methods that are recommended (+++) and suitable (++) for confirmation of clinical cases and certifying freedom from peste des petits ruminants

Diagnostic method	Purpose				
	Target	Case confirmation	Population freedom	Immune status	International trade
IC- ELISA	Viral protein	+++			
RT-PCR	Viral genome	+++			
Virus isolation	PPRV	++			
VNT	Antibodies		+++	+++	+++
C-ELISA	Antibodies		++	+++	

### Search strategy and selection criteria

**Fig. 1:**
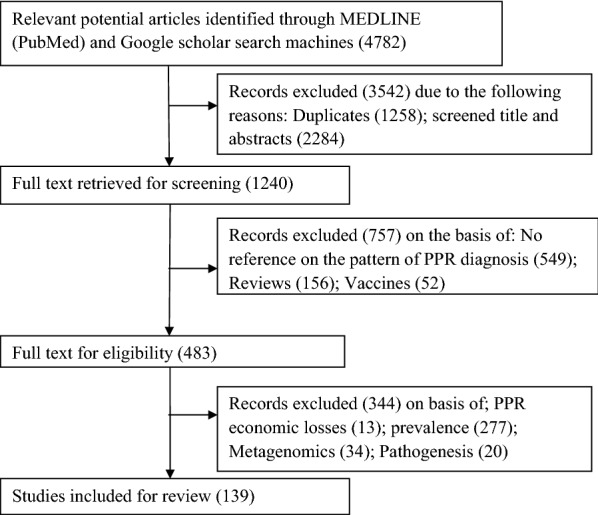
Flow diagram for the review process

The MEDLINE (PubMed) and Google scholar search machines were used as source of the peer-reviewed articles included in this review. The articles were selected using keywords combined by Boolean operators (peste des petits ruminants OR PPR OR *Peste des petits ruminants virus* OR PPRV OR diagnosis OR diagnostic*AND (PPR diagnosis). All searches on PPR diagnosis were performed in 2 years. Only 142 articles out of 4782 written in English from the first description of PPR in 1942 met inclusion criteria as shown in PRISMA flow diagram (Fig. 1).

### Susceptible animal species and transmission of peste des petits ruminants

PPRV infects domestic as well as wild ruminants with goats and sheep being the most susceptible domestic animals and which also serve as primary hosts. The disease has been reported to be more severe in goats than in sheep, although this claim still lacks scientific proof [35]. Transmission of PPRV occurs through direct contact with infected animals, inhalation of aerosol (expectorate), or contacts with lacrimal secretions, nasal exudates, saliva and faeces.

Studies have shown that both camels and suids are susceptible to PPRV infection and develop clinical disease [36, 37]. The role of wildlife animals and domestic Artiodactyls in the epidemiology of PPR is unknown or insufficiently understood [38]. Infections of various wildlife species including African buffalo (*Syncerus caffer*) and many antelope species occur apparently subclinical [38, 39] but the only confirmed reports of disease in African wildlife have occurred under captive or semi-free range conditions [40, 41]. According to previous studies, animals that recover from PPRV infections develop life-long immunity [42, 43]. Because of its immunosuppressive effect, PPRV infections are usually accompanied by secondary infections thereby complicating clinical diagnosis.

### Clinical manifestation

**Fig. 2:**
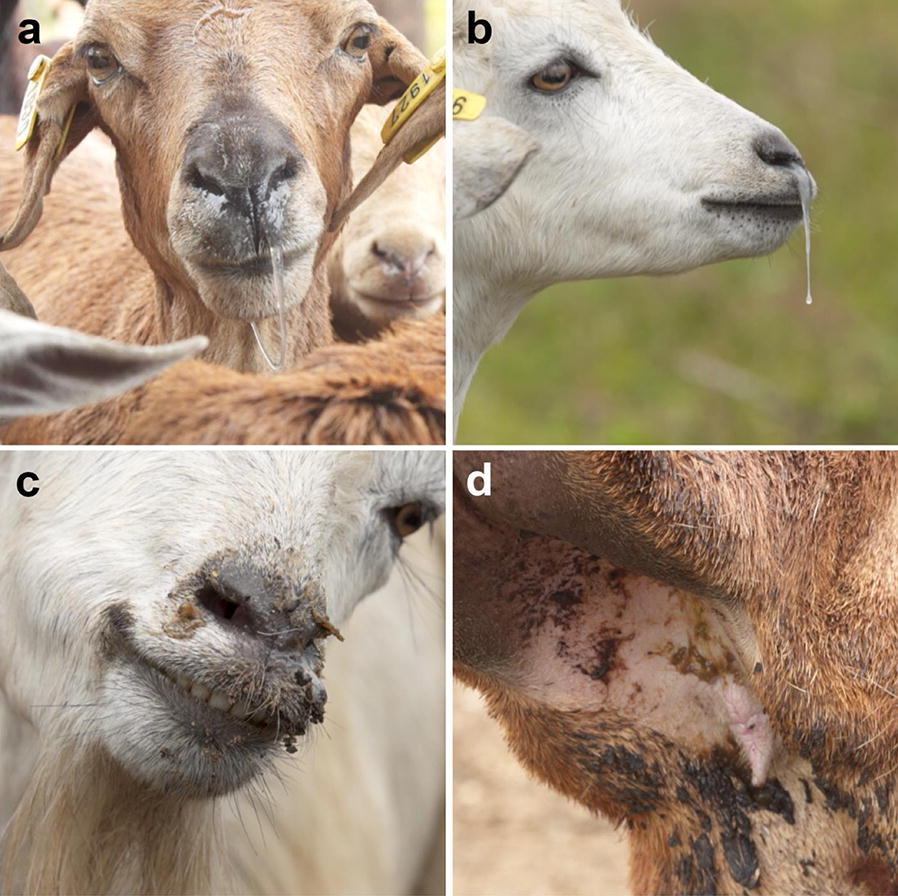
Clinical signs in goats and sheep confirmed with *peste des petits ruminants virus* infection in a farm located in Tanga, Tanzania. Nasal discharges in **a** a sheep and **b** a goat, **c** dried-up purulent nasal discharges in a goat, and **d** diarrhoea in a sheep

It takes 3–4 days before onset of clinical signs. During this incubation period, PPRV replicates in the draining lymph nodes of the oropharynx followed by spreading (via blood and lymph) to other tissues and organs including the lungs resulting in a primary viral pneumonia. The acute stage of disease is characterised by high body temperature (39.5 to 41 °C) which may last for 3–5 days [44]. Other signs are depression, anorexia, dry muzzle, excessive salivation, lachrymal discharges and serous nasal discharge, which gradually turn mucopurulent (Fig. 2). The affected animals develop papules in the oral cavity, which become erosive and necrotic. In severe cases, these necrotic lesions occur concurrently with fibrin deposits on the tongue [24, 35, 45]. In the later stages, there is diarrhoea and cough with labored abdominal breathing. Terminally, the animal may become dyspnoeic, progressively lose weight and eventually dies. In mild infections, self-cure occurs after 10–15 days of infection. Protective immunity responsible for self-recovery is attributed to infection-induced antibodies against the haemagglutinin (H) and fusion (F) proteins [46, 47], although most of the neutralizing antibodies are directed against the H protein [48]. Nucleoprotein (N) is the most abundantly transcribed gene in the host cells. For this reason, the H and N proteins are the two most preferred PPRV targets for the development of vaccine and immunodiagnostics, respectively [49, 50].

### Epidemiology of peste des petits ruminants

The development of specific and sensitive molecular and serological techniques have improved the diagnostic precision for PPR since not all cases of PPR can be distinguish from rinderpest, pneumonic pasteurellosis and contagious caprine pleuropneumonia, based on clinical sigs [4]. Based on previous similar outbreaks of the disease in Senegal and Guinea in 1871 and 1927, respectively, it was believed that PPR might have been existing much earlier than previously thought [9, 10]. The disease spread subsequently to the neighbouring African countries like Nigeria and Ghana [51]. Until early 1980s, definite outbreaks of PPR were reported from different parts of West Africa [42, 52] and it was regarded as a disease of West African countries. However, it was later realised that the disease spread beyond West Africa with cases being recorded in Sudan [53]. In the northern part of Africa, PPR was reported in Morocco in 2008 and later Egypt, Algeria and Tunisia have also reported PPR [54–56]. Globally, PPR affects about 70 countries in Africa, Asia and the Middle East [57]. Out of 70 countries that have either reported PPR infection to the OIE or are suspected of being infected, more than 60% are in Africa (except southern Africa). Other infected countries are in Asia (South-East Asia, China, South Asia and Central Asia/West Eurasia including Turkey) and the Middle East [13, 14, 57]. PPRV lineages I and II have been found exclusively in western and central Africa; lineage III is common to eastern Africa and the southern part of the Middle East. Lineage IV is found in Southeast Asia, Middle East and North Africa [23, 44, 58–60]. Incursions of PPRV lineages have been also reported, for instance lineage II and IV were found in East Africa and lineage IV in Ethiopia [12, 61–64]. Recent outbreaks of PPR in Bulgaria, Georgia and the Marmara region in Turkey increase the threat to Europe [65–67]. The spread of PPR beyond its usual boundaries is attributed to cross-border movements of animals and animal products, which are being promoted by trade, nomadic lifestyle, tourism and migration of wild animals [9, 59, 68].

### PRRV as a target for diagnostics and vaccine development

Like other members of the genus *Morbillivirus*, PRRV is enveloped, pleomorphic with the particles diameter ranging from 400 to 500 nm [1]. The genome is a linear, non-segmented negative sense single stranded RNA, which is15,948 nucleotides long. There are six genes i.e. 3′-*N, P, M, F, H, L*-5′, which constitute the genome. Each of these genes codes for a distinct structural protein and each of these proteins bear the acronym of the respective gene of origin; nucleoprotein (N), phosphoprotein (P), matrix protein (M), fusion protein (F), haemagglutinin protein (H) and large polymerase protein (L). Besides coding for the phosphoprotein, the *P* gene also codes for two non-structural proteins designated C and V. The C and V proteins are generated through alternative start codons (leaky scanning) and RNA editing, respectively [69]. It has been shown that the N protein is abundant in infected cells and highly immunogenic. On the other hand, given its abundance and antigenic stability, the N protein has been a preferred candidate antigen for development of PPRV immunodiagnostics [70, 71]. The P protein is a co-factor for viral replication and transcription in conjunction with the L protein, which is an RNA-dependent RNA polymerase. The M constitutes the inner coat of the viral envelope and acts as a bridge connecting the surface glycoproteins (F and H) to the ribonucleoprotein core. The H protein mediates attachment of virions to the host receptors whilst F protein induces fusion of the viral membrane with the host cell membrane in order to enter host cells [70]. Then on-structural proteins play several functions including viral RNA synthesis, virulence determination and modulation of RNA-dependent RNA polymerase activity and blockade of interferon signaling.

PPRV exists as a single serotype, but is divided into four distinct genetic lineages [72]. The development of necessary tools for PPRV control, including vaccines and diagnostics, greatly relied on detailed insights into the PPRV genome and protein constituents; and on this same basis the virus is now classified into lineages. Structurally, the N protein is divided into four regions I, II, III and IV of amino acids sequences, 1–120, 121–145, 146–398 and 421–525, respectively [49]. Owing to high immunogenicity of regions I and II, these could be targeted for improvement and development of immunoassays. On the other hand, based on H protein mapping of the functional domain, two regions are the most immuno-dominant epitopes (263–368 and 539–609) and are diverse among all the members of Morbilliviruses with significant potential for development of a DIVA vaccine that could differentiate infected from vaccinated animals [47].

### Tentative diagnosis

Presumptive diagnosis of PPR is based on clinical signs presented in living animals and postmortem lesions. Such diagnosis in PPRV endemic zones could play an important role in early warning in disease-symptomatic surveillance when coupled to digital diagnostic technology [73–75]. However, definitive laboratory diagnosis of PPR is the key to achieving accurate result because PPRV infections manifest similar clinical picture with other diseases such as bluetongue, contagious caprine pleuropneumonia, capripox and foot-and-mouth disease [4, 23, 76].

### Virus isolation

PRRV isolation using primary cells (bovine, ovine and caprine kidney and lung epithelial cells) requires multiple, sequential blind passages and takes up to weeks in culture before the development of any cytopathic effect [42, 77]. The quality of primary cells is not guaranteed due to the presence of endogenous virus and there is considerable batch to batch variation (Table 3). The infection efficiency of PPRV in primary cultures is up to 100–1000 times less than that of the lymphoid cells expressing signaling lymphocyte activation molecule (SLAM) [78, 79]. Thus, non-lymphoid cells expressing this recombinant protein, are used to isolate and propagate PPRV efficiently [79]. However, transformed marmoset B-lymphoblastoid cells (B95a) derived from Epstein-Barr virus, are more sensitive and support better growth of PPRV lineage IV compared to Vero cells. Virus isolation is expensive and time consuming, thus, it cannot be deployed for routine diagnostic, but it can only be used as gold standard for further disease confirmation and in research studies. Thus, establishment of cell lines with high infection efficiency to PPRV will be of help in confirming viable PPRV in the last phase of global PPRV eradication (Table 3).

**Table 3:** Progress towards the development of suitable platforms for PPRV isolation, maintenance and production of biosafe antigen

Platform	Strength (s)	Limitation (s)	References
Primary cell culture	Cheap and easily accessible	Variations in batches and low quality due to the presence of endogenous viruses	[53, 77, 80–82]
Vero cells	Easy to maintain in culture	Low infection efficiency compared to lymphoid cells	[42, 81, 83]
Madin-Darby bovine kidney epithelial cell line (MDBK)	Suitable for PPRV isolation.	Requires multiple sequential blind passages for visible cytopathic effect	[84]
MDBK-nectin-4 cell line	Rapid for clinical isolation of PPRV	Only limited to Nectin-4 and high overhead cost	[85, 86]
Baby hamster kidney (BHK-21)	Suitable for growth kinetics of PPRV	PPRV replicates at relatively lower titers in BHK-21 cells	[87, 88]
Vero-SLAM	Highly efficient for PPRV isolation	Prone to fungal and bacterial contaminations	[79, 89, 90]
Vero dog SLAM-L protein (VDS-L)	Produces biosafe antigens in low level biocontainment	Prone to fungal and bacterial contaminations	[91]
Alpine goats	Suitable for in vivo pathological studies	Require high level containment	[92]

### Paradigm shift in peste des petits ruminants diagnostic assays

For PPR diagnosis, a plethora of serological and molecular assays have been developed with continuous on-going improvements. The assays detect PPRV antigens, nucleic acid or PPRV induced antibodies [93]. These assays include agar gel immunodiffusion (AGID), counter-immunoelectrophoresis (CIE), enzyme linked-immunosorbent assay (ELISA),reverse transcription polymerase chain reaction (RT-PCR) either gel-based or real time, or reverse transcription loop mediated isothermal amplification (RT-LAMP), reverse transcription recombinase polymerase amplification assays (RT-PRA), immunochromatographic lateral flow devices (IC-LFDs), luciferase immunoprecipitation system (LIPS) and pseudotype-based assays. They vary in their technical demands, but all require a laboratory with exception of IC-LFDs, and possibly RT-LAMP and RT-RPA (Table 4). The AGID and CIE assays are less sensitive at the early stages of infection where antigen levels are below detectable threshold and could only detect 42.6% of ante-mortem and necropsy specimens [94]. Thus, progress was made to replace them with assays that are more sensitive and specific, such as ELISA, immunochromatographic assays and nucleic acid-based assays [95–98]. ELISA employs an enzyme–substrate reaction for the detection of antigen–antibody interactions. They are suitable for screening large sample sizes and better documentation of evidence-based clinical samples status. Later on, a high sensitive immunocapture enzyme-linked immunosorbent assay (IC-ELISA) was developed based on conventional monoclonal antibodies (MAbs)and it demonstrated diagnostic sensitivity of 10^0.6^ TCID_50_ [74, 95, 99]. Again, sandwich-ELISA and dot-ELISA based on conventional antibodies were developed and they have been in use since 2002. Dot-ELISA when was compared for its relative diagnostic sensitivity and specificity with routinely used sandwich-ELISA were 82% and 91% respectively, for the diagnosis of PPR [100]. However, dot-ELISA could serve as simple field test to screen clinical samples from suspected PPR cases. In comparison with commercial IC-ELISA kit, sandwich ELISA exhibited 88.9% and 92.8% relative diagnostic sensitivity and diagnostic specificity, respectively [101]. Although the sensitivity of the dot-ELISA is lower, it can also be used in combination with other assays such as LIPS, pseudotype-based assays and nucleic acid-based tests in laboratories where resources are limited. The gel based RT-PCR assays serve to detect viral nucleic acid with high sensitivity and accuracy regardless of being labor intensive, time-consuming and prone to high risk of cross-contamination. Alternatively, real-time RT-PCR assays detect and quantify PPRV present in clinical samples in real time [102]. The high cost of the equipment and technical demands impede its utility in low-income countries. In low-income countries, potent, inexpensive field-deployable diagnostic tools are prospect for use in the prevention and control measures of PPR. Recently developed lateral flow devices based on conventional antibodies have rejuvenated hopes in the least developed countries for rapid detection of PPRV [6, 26]. Despite the pen-side versatility of some lateral flow devices, their sensitivities were not able to detect PPRV in clinical samples with a low virus load as sensitively when compared to IC-ELISA, LIPS and nucleic acid-based diagnostic tools. Detection of serum antibody is also not effective because all assays based on detection of PPRV antibody could not differentiate infected animals from vaccinated animals. Recombinant antigen-based assays are of value during post vaccination evaluation and in the last phase of eradication where free PPRV diagnostic tools are required [96, 103–108]. On the other hand, a battery of potential field-based diagnostic tools have been developed and introduced for use in the diagnosis of PPR (Table 4).

**Table 4:** Demonstration of peste des petits ruminants diagnostic spectrum and prototype assays undergoing development

Diagnostic technique	Reliability		References
	Strengths	Limitation (s)	
Tentative diagnosis	Less costly	Unreliable due to presence of PPR related diseases	[73, 109]
Virus culture and isolation	Discerns active infections	High overhead cost	[77, 79, 110]
Virus neutralisation test (VNT)	It is specific and able to discern PPRV exposure	Cannot be used as DIVA test	[53, 111]
Agar gel immunodiffusion (AGID)	Simple and cheap	Low sensitive and is affected by prozone effect	[94, 112]
Counter-immunoelectrophoresis (CIE)	The test is fast, simple and cheap	Not free from prozone effect	[94, 112]
Enzyme-linked immunosorbent assays (ELISA)	Suitable for routine diagnosis on large scale	Low sensitive compared to PCR	[95, 96, 113–115]
Haemagglutination (HA) test	Simple to perform and it is inexpensive	Non-specific	[116–118]
Haemagglutination inhibition (HAI) test	Fast and relatively easy to perform and easy to standardise	Works best with human blood group‘‘O’’	[119, 120]
Immuno-peroxidase test	Test is easy to perform	Test is less sensitive compared to RT-PCR	[109]
Fluorescent antibody test (FAT)	The test is highly specific and able to detect active infection	High overhead cost and impracticable in the field setting	[116]
Immunofiltration test	Pen-side test and serves to screen large sample size	Less sensitive compared to ELISA	[105]
Immunochromatographic test	Rapid and does not require instrumentation	Less sensitive compared to IC-ELISA	[26]
Luciferase immunoprecipitation system tests	Highly sensitive for sero-surveillance	Not DIVA test	[121]
Pseudotype-based assays	No need of sophisticated facility	Technically demanding test	[122]
Quantum dots-lateral flow immunoassay strips	Very rapid test and highly sensitive	Limited to previous exposure	[27]
Surface Plasmon resonance-biosensor	Ultrasensitive diagnostic tools	Expensive and technically demanding	[123, 124]
Reverse transcription polymerase chain reaction (PCR)	Highly sensitive and accurate	High maintenance cost	[97, 125]
Reverse transcription loop-mediated isothermal amplification	Highly sensitive, cheap and rapid for pen-side test	Requires many primers	[29]
Microarray	It allows multiple virus screening	Less sensitive compared to PCR	[21, 126]
Reverse transcription recombinase polymerase amplification	Point of care diagnostics following miniaturisation	Sensitivity is low compared to RT-PCR	[32, 127]
Sequencing platforms	Highly accurate for aetiologic agents confirmation	Costly and require expertise	[128–130]
Oxford nanopore MinION sequencers	Rapid and accurate for genomic surveillance in field settings	Requires extra efforts for monitoring signal to noise ratio in base detection	[33, 129]

### Recent advances in peste des petits ruminants field-deployable diagnostic assays

#### Immunochromatographic lateral flow test

A novel pen-side diagnostic tool for diagnosis of PPR was developed at The Pirbright Institute (Pirbright, UK) in 2014. This lateral flow immunochromatographic assay is based on the specificity and affinity of conventional monoclonal antibody (MAb) C77 that was prepared using hybridoma cells technology in a miniPerm bioreactor and purified on a protein G HiTrap column [26]. The C77 MAb recognises the PPRV H protein and has been previously used in a prototype pen-side test for PPRV and rinderpest [6, 26]. In principle, the MAb C77 serves as the antigen fishing reagent on the chromatographic test strip and detection reagent that is labeled with colloidal gold-red. The performance of this diagnostic assay was evaluated in the laboratory and under field conditions on a superficial sample (ocular or nasal swabs). The test showed a sensitivity and specificity of 84% and 95%, respectively, relative to RT-PCR and detected as little as 10^3^ TCID_50_of cell culture-grown PPRV. The test could detect PPRV in swabs from animals as early as 4 days post-infection at a time when clinical signs were minimal. The IC-LFD kit is a prospect for field diagnosis of PPR and is being manufactured by Foresite Diagnostics Ltd (Sand Hutton, York, UK). The availability of this field-deployable diagnostic tool in developing countries will improve the diagnostic capacity for PPR. This will lead to early detection, which will significantly reduce the negative impact of PPR. Furthermore, this method could be utilised in the field without the need for expensive equipment, removing the requirement for its operation in a well established laboratory. However, case confirmation is essential during an outbreak of PPR. In such situation, field friendly, rapid and accurate nucleic acid-based diagnostic tools (RT-LAMP, RT-RPA and Oxford nanopore MinION sequencers) could be deployed.

#### Quantum dots-lateral-flow immunoassay strip

Recently, a fast and ultrasensitive quantum dots lateral flow immunoassay strip was established at the State Key Laboratory of Agricultural Microbiology in China to detect anti-PPRV antibodies. In this assay, N protein of PPRV is immobilised on the detection zone of the test strip and luminescent water-soluble carboxyl-functionalised quantum dots were used as signal output and were conjugated to streptococcal protein G. The performance of the test is extraordinary compared to C-ELISA and the IC-LFD for PPR serum IgG antibody detection [27]. The test is rapid, sensitive and suitable for on-site, point-of-care diagnosis and post vaccination evaluation of PPRV. This test cannot be used for early detection of active infection where only IgM and viral particles are present in circulation. Alternatively, nucleic acid-based tests or PPRV antigen detection methods could be used to assess during disease outbreaks.

#### Reverse transcription loop-mediated isothermal amplification assay

A novel inexpensive RT-LAMP provides an isothermal method to amplify viral RNA without the requirement of expensive specific thermal cycler [29, 30]. Moreover, RT-LAMP reagents can be stored at ambient temperature for at least 2 weeks. The RT-LAMP reaction could be performed in an inexpensive water bath, dry bath, or heat block and the reaction results could be directly distinguished through color change or formation of the precipitate by the naked eye or alternatively via agarose gel electrophoresis or real-time turbid meter. Reverse transcription loop mediated isothermal amplification assays have been developed for the diagnosis of PPR based on M and N genes of PPRV with higher sensitivity than RT-PCR [28]. This sensitive, inexpensive and streamlined method can be more readily used in developing countries that do not have access to high technology laboratories. However, in each RT-LAMP assay, primers must be specifically designed to be compatible with the target nucleic acid sequences, which may discourage researchers. In addition, the RT-LAMP assay requires six primers and has unsatisfactory reliability in detection of highly variable viruses. An alternative field deployable recombinase polymerase amplification assay was developed targeting viruses of veterinary importance [32].

#### Reverse transcription recombinase polymerase amplification assay

In the advancements of the novel point-of-care molecular tests in recent years, RT-RPA assay was developed and coupled to a lateral flow. This assay is used for rapid detection of different viruses and parasites of veterinary and public health importance [32, 127].The assay demonstrated a pen-side usefulness for rapid detection of pathogens such as PPRV, *Foot*-*and*-*mouth disease virus*, *Orf virus*, *Bovine viral diarrhoea virus* and *Leishmania spp* [32]. Generally, the assay uses recombinase, single strand binding protein, strand displacing DNA polymerase and a fluorescent probe. Then, the lateral-flow strips are coupled in the detection system. These assays are highly specific for detection of PPRV as there is no cross-reaction with *Foot*-*and*-*mouth disease virus* and *Orf virus*, which may cause similar clinical signs to PPRV in small ruminants, indicating the potential of being a novel testing tool for differential diagnosis [32]. Although the sensitivity of RPA is lower than for RT-PCR, some advantages of the RT-RPA assay over RT-PCR assay make it rather attractive. Firstly, reaction mixtures are pre-made pellets and provided in vacuum-sealed pouches, which can be kept at room temperatures for several days. This would save on cold chain costs and facilitate on-site diagnosis of PPR in the field. Secondly, the reaction can be performed in a water bath at a temperature of 37 to 45 °C for a maximum of just 20 min. The RT-RPA assay is rapid compared to RT-LAMP and it does not require expensive equipment and the results are read with the naked eye in less than 25 min. However, virus genetic sequences analysis could not be determined by aforementioned diagnostic tools in field settings to match with the plasticity of RNA viruses including PPRV. The Oxford nanopore MinION sequencers may be of choice in such situation.

#### Oxford nanopore MinION sequencers

The Oxford nanopore MinION technology brings rapid comprehensive detection, diagnostics, and bio-surveillance of emerging infectious diseases to extremely remote and physical challenging geographical landscapes, completely detached from the traditional physical building. This technology has been used in arbovirus surveillance and during Ebola and Zika outbreaks [34, 129]. This technology is non-PCR-based tool for meta-transcriptomic detection of RNA virus from the clinical samples using Oxford nanopore MinION sequencers. In principle, clinical samples are processed in the Biomeme’s bulk nucleic acid extraction developer kit. There are few challenges in applying Oxford nanopore MinION sequencing to diagnosis of infectious diseases, these include; high host’s nucleic acid to pathogen ratio and low quality nucleic acid in the sample. It is, therefore, very important to carefully design, develop and optimise diagnostics pipelines before attempting to apply them to clinical samples. This diagnostic platform may provide the capacity for genomic surveillance of PPRV as well as other infectious diseases in resource-limited settings in real time.

### Rational integration of diagnostic tools for diagnosis of peste des petits ruminants

Despite of increased development and use of PPRV novel field-deployable diagnostic tools, diagnostics are not being integrated into disease control optimally. A comprehensible integration of diagnostic tools is essential in PPRV endemic areas. The efficient integration of diagnostics may be influenced by a multitude of factors including the existence of co-infections, clinically closely related diseases and asymptomatic cases in both wildlife and domestic animals. It is further influenced by the availability of appropriate technology and access to diagnostics, test characteristics, veterinary infrastructure and the experience and knowledge of the veterinary service providers in resource-limited settings. Regardless of the varying sensitivity and specificity of PPR diagnostic tests and prevalence of disease, it is clear that diagnostics play a valuable and critical role in early detection of PPR in infected animals with disease and those at risk of developing the disease.

**Fig. 3:**
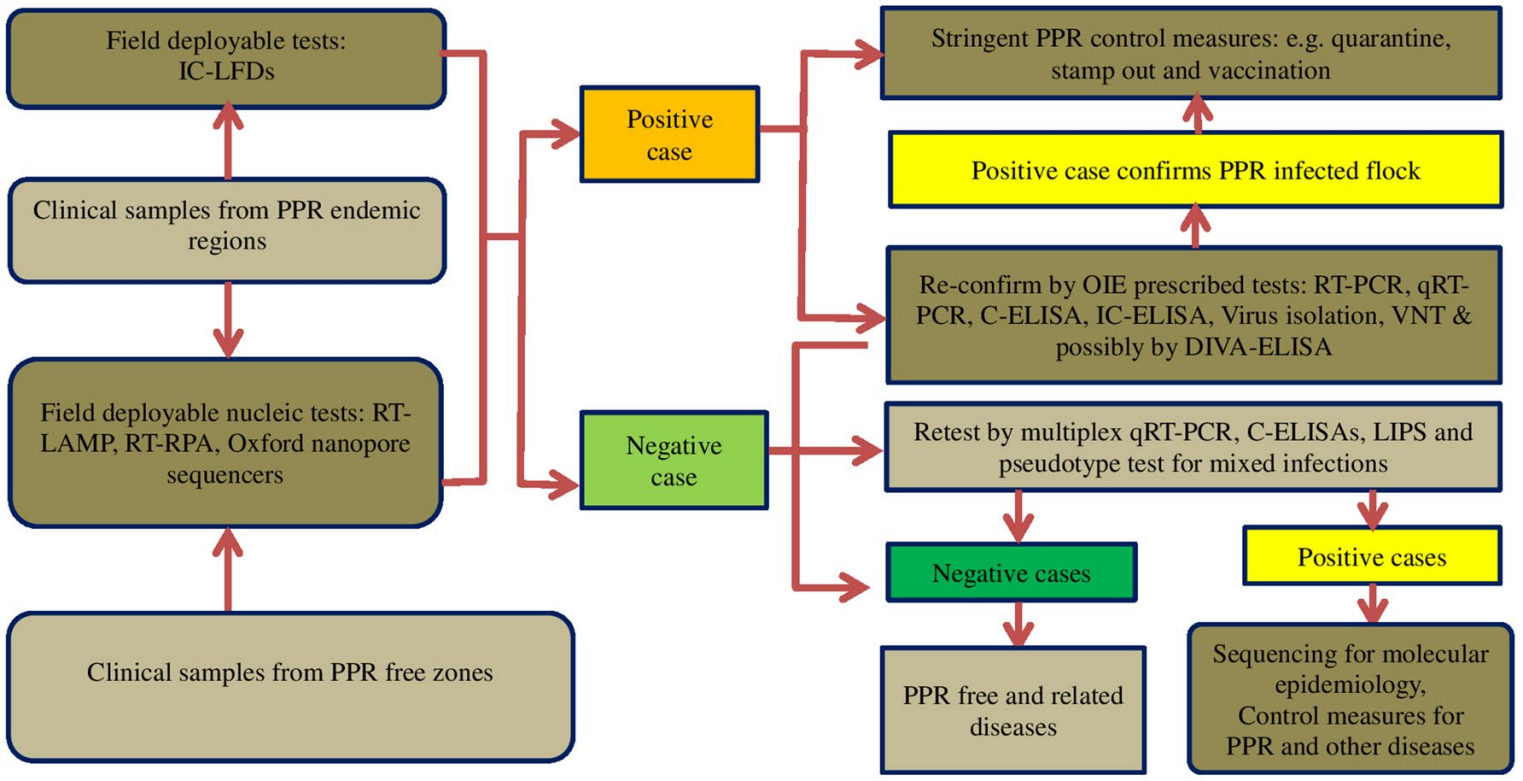
Rational integration of diagnostic tests for rapid and correct identification of peste des petits ruminants

Most of PPR field deployable IC-LFDs have lower sensitivity than IC-ELISA, LIPS and nucleic acid-based tests, but they are still useful tests on the PPR-battlefield in extremely remote settings, away from traditional veterinary laboratories. For instance, in the entire flock, if a less sensitive test has scored some animals’ positive for PPR, the herd would be grouped as infected or at risk of developing a disease. Therefore field level diagnosis should be institutionalised for early screening and transportation of PPRV suspected samples from rural remote areas and ecosystem containing susceptible wildlife. In turn, early control measures will be put in place to prevent further spread of PPRV to neighboring flocks or a distant PPRV free zone (Fig. 3). A good example is a death toll of the saiga antelope; whereby a rapid diagnostic tool (PPR Rapid—BD SL Pirbright UK) was deployed with subsequent confirmation by PCR and sequencing [17].

The characteristics of an ideal diagnostic test include accuracy wherever used; heat-stable reagents with an extended shelf life; portability; minimal technical skills for operation; rapid, sensitive, and specific results; on-demand testing capability or minimal batch sizes; cost effective tests; and suitable for a broad range of clinical samples [131]. The current PPRV antigens and nucleic acids based tests meet some, but not all, of these standards. This has led to the development of automated diagnostic tools. The newer automated tests using nanotechnology that no longer require the addition of multiple reagents are being used for point-of-care diagnosis. Therefore, for recognition of PPRV during early phases of the disease and for clinical samples that gave equivocal results in other tests and require re-confirmation, highly sensitive nucleic-acid-based diagnostic tests could be used [6]. A good number of these tests are available commercially including IC-LFDs, RT-LAMP and Oxford nanopore MinION sequencers and their uses continue to increase logarithmically and the cost of instruments and their assays continue to decrease and are becoming of high value in resource-limited nations.

Moreover, in cases of co-infections, the multiplex assays utilizing real-time amplification methods may be of value for simultaneous detection of multiple viral infections in PPR infected animals as some viruses are preferentially replicating in PPR infected hosts [132]. These assays may also detect the nucleic acid of viruses that are previously unrecognised and/or not cultivatable in vitro. Inevitably, high mutation rates in RNA viruses including PPRV can be rapidly identified using nucleic acid amplification coupled with sequencing platforms such as Oxford nanopore MinION sequencers, to curb highly emerging or reemerging virulent strains. In resource-limited countries, conventional methods are more readily replaced in virology because the tissue culture based virology methods are costly and generally less sensitive than newer molecular methods.

Civil unrest, global climate and environmental change from hurricanes, flooding, and earthquakes have a dramatic influence on the frequency of certain diseases in new locations. A minute or dramatic change in the environment can have a significant impact on the spread of diseases including PPR. These catastrophes including droughts or wars may lead to humans’ movements from place to place with their belongings, including animals. In such situations, contagious diseases like PPR may spread very rapidly. Therefore to curb PPR spread and control animals’ movements, highly sensitive nucleic acid-based field-deployable diagnostic tools are critical tools in keeping up with continuously changing disease dynamics. In addition, prompt outbreak identification is central to controlling the spread of highly contagious diseases like PPR, but recognizing that an outbreak has occurred can be difficult. Most molecular methods to detect different viral strains require that specimens are sent to distant reference laboratories, with confirmation of an outbreak possibly requiring days to weeks in resource-constrained countries. The development and use of both field deployable immunoassays and molecular diagnostic tools such as IC-LFDs, RT-LAMP, RT-RPA and Oxford nanopore MinION sequencers may play a significant role in controlling disease outbreaks in extremely remote areas in which transportation of clinical samples in optimum cold chain is unreliable (Fig. 3).

### Prospects of nanobodies for use in immunoassays

In the early 90’s, Hamers-Casterman and her colleagues made a remarkable discovery where they found a structurally different kind of antibodies which are part of the humoral immune response in the serum of camelids [133]. The heavy chain-only antibodies (HCAbs) in camelids or similar molecules in shark (Ig-NAR) are devoid of light chains [134]. The antigen binding domain of Ig-NARs or HCAbs involves a single domain only, referred to as V-NAR when derived from Ig-NARs and VHHs (nanobodies) when derived from HCAbs. The nanobodies have proven to be powerful tools in diagnostics due to their unique characteristics [135]. In particular, the recombinant expression of nanobodies in microbial systems and straightforward purification using His-tag by immobilised metal affinity chromatography makes their purification easy and very cheap. In contrast, traditional MAbs (used in virus detection) need more support costs and they are difficult for massive production compared to nanobody generation strategies [136, 137]. Furthermore, nanobody proteins are robust against thermal denaturation, which obviates a cold chain for transport and storage. Interestingly, despite that nanobodies recognise their cognate antigen via one single domain-only, they still achieve a high affinity and specificity. In addition, the convex paratope of the nanobodies comprising three antigen binding loops or complementary determining regions prefer to interact with a concave surface on the antigen, an architecture that is not antigenic for classical antibodies [134]. Therefore, the use of nanobodies may circumvent binding interference caused by the host’s antibody response. Thus, nanobodies should detect both free antigens as well as those bound by host antibodies, which would make nanobodies based diagnostic tests rather attractive [138]. In contrast, the large size of MAbs prevents them from reaching cryptic epitopes andhost IgG molecules might as well conceal the epitopes from the MAbs employed in the diagnostic test [139]. The use of the nanobody coupled with lateral flow device may accelerate the development of cost effective, highly sensitive, specific and rapid immunoassays. In the light of global PPR eradication, a cost-effective, multiplex (multi-disease) diagnostic test would be very useful for concurrent bio-surveillance of PPR and similar infectious diseases such as contagious caprine pleuropneumonia, bluetongue, contagious ecthyma and foot-and-mouth disease. These multiplex assays may also be indispensable in all phases of PPR eradication operation to rule out mixed infections.

## Conclusions

Peste des petits ruminants incidence is growing at an alarming rate worldwide and continues to undermine the economic activities of the poorest farmers and threatens biodiversity. However, in resource-limited setting, expensive sophisticated diagnostic tools are at risk of becoming redundant, due to insufficient funds for consumables, maintenance and expertise. Thus, the availability and distribution of cost effective field-deployable diagnostic tools in developing countries will improve diagnostic capacity and early containment of PPR. Field deployable and OIE prescribed laboratory based diagnostic tools have inherent strengths and weaknesses, thus optimal amalgamation is essential for rapid and accurate diagnosis of PPR.

## Data Availability

The dataset analyzed during the current study are available from the corresponding author on reasonable request.

## Abbreviations

AGID: agar gel immunodiffusion
CIE: counter-immunoelectrophoresis
C-ELISA: competitive enzyme-linked immunosorbent assay
ELISA: enzyme-linked immunosorbent assay
FAO: Food and Agriculture Organisation
HCAbs: heavy chain-only antibodies
IC-ELISA: immunocapture enzyme-linked immunosorbent assay
IC-LFD: immunochromatographic lateral flow devices
IgG: immunoglobulin G
IgM: immunoglobulin M
LIPS: luciferase immunoprecipitation system
MAbs: monoclonal antibodies
OIE: World Organisation for Animal Health
PPR: peste des petits ruminants
PPRV: *peste des petits ruminants virus*
RT-LAMP: reverse transcription loop mediated isothermal amplification
RT-PCR: reverse transcription polymerase chain reaction
RT-RPA: reverse transcription recombinase polymerase amplification assay
SACIDS: Southern African Centre for Infectious Diseases Surveillance
SACIDS-ACE: SACIDS-Africa Centre of Excellence for Infectious Diseases of Humans and Animals in East and Southern Africa
SLAM: signaling lymphocyte activation molecule
VHH: nanobody
VNT: virus neutralisation test
